# Performance of an Artificial Multi-observer Deep Neural Network for Fully Automated Segmentation of Polycystic Kidneys

**DOI:** 10.1007/s10278-017-9978-1

**Published:** 2017-05-26

**Authors:** Timothy L. Kline, Panagiotis Korfiatis, Marie E. Edwards, Jaime D. Blais, Frank S. Czerwiec, Peter C. Harris, Bernard F. King, Vicente E. Torres, Bradley J. Erickson

**Affiliations:** 1 Department of Radiology, Mayo Clinic College of Medicine, 200 First St SW, Rochester, MN 55905 USA; 2 Division of Nephrology and Hypertension, Mayo Clinic College of Medicine, Rochester, MN USA; 3 Otsuka Pharmaceutical Development & Commercialization Inc., Rockville, MD USA

**Keywords:** Autosomal dominant polycystic kidney disease, Deep learning, Magnetic resonance imaging, Planimetry, Segmentation, Total kidney volume

## Abstract

Deep learning techniques are being rapidly applied to medical imaging tasks—from organ and lesion segmentation to tissue and tumor classification. These techniques are becoming the leading algorithmic approaches to solve inherently difficult image processing tasks. Currently, the most critical requirement for successful implementation lies in the need for relatively large datasets that can be used for training the deep learning networks. Based on our initial studies of MR imaging examinations of the kidneys of patients affected by polycystic kidney disease (PKD), we have generated a unique database of imaging data and corresponding reference standard segmentations of polycystic kidneys. In the study of PKD, segmentation of the kidneys is needed in order to measure total kidney volume (TKV). Automated methods to segment the kidneys and measure TKV are needed to increase measurement throughput and alleviate the inherent variability of human-derived measurements. We hypothesize that deep learning techniques can be leveraged to perform fast, accurate, reproducible, and fully automated segmentation of polycystic kidneys. Here, we describe a fully automated approach for segmenting PKD kidneys within MR images that simulates a multi-observer approach in order to create an accurate and robust method for the task of segmentation and computation of TKV for PKD patients. A total of 2000 cases were used for training and validation, and 400 cases were used for testing. The multi-observer ensemble method had mean ± SD percent volume difference of 0.68 ± 2.2% compared with the reference standard segmentations. The complete framework performs fully automated segmentation at a level comparable with interobserver variability and could be considered as a replacement for the task of segmentation of PKD kidneys by a human.

## Introduction

A particular section of machine learning, known as deep learning, is currently enjoying its renaissance in the area of artificial intelligence [1]. For computer vision tasks, the primary motivation of deep learning techniques is the biomimicry of the human visual system, allowing computers to learn from experience and formulate an understanding in terms of a hierarchy of concepts. In the field of medical image processing, deep learning approaches are providing computational solutions to a wide range of automation and classification tasks [2]. For instance, deep learning techniques have been used in organ [3] and tumor segmentation tasks [4], as well as tissue and tumor classification [5, 6]. The fundamental difference of deep learning methods is that they take a unique approach to solving classical image processing tasks by allowing the computer to identify image features of interest. This is in contrast to traditional machine learning that requires predefining the features of interest (e.g., image edges, intensity, and/or texture). Based on the successes of deep learning techniques, we sought to explore their potential in solving the difficult task of segmenting the kidneys of patients affected by autosomal dominant polycystic kidney disease (ADPKD).

In ADPKD, these phenotypic differences include renal size (e.g., renal volumes can vary from ~200 ml to more than 7000 ml), shape, and composition (e.g., appearance of the border of the kidneys in MR images has highly variable signal intensities resulting from whether the border is composed of simple and/or complex cysts, varying degrees of fibrosis, or healthy renal parenchyma). The natural course of ADPKD is highly variable and is characterized by progressive enlargement of cysts within the kidneys and is a leading cause of end-stage renal disease (ESRD) [7–10]. Total kidney volume (TKV) has become the main image-based biomarker for following ADPKD progression at early stages of the disease [11–15]. Imaging methods such as ultrasound (US), computed tomography (CT), and magnetic resonance imaging (MRI) are employed to diagnose, monitor, and predict outcomes for patients affected by ADPKD [16–19]. MRI has become the imaging modality of choice due to its superior soft tissue contrast, non-ionizing radiation, and accuracy. Current methods to manually measure TKV using MR images include volume calculation by the ellipsoidal method [20], stereological approaches [21], and planimetry tracings [22, 23].

Due to the large time requirement of manual tracing, automated approaches to segment kidneys are desirable. However, segmentation of ADPKD kidneys is challenging due to a number of factors. For instance, the shapes of the kidneys are highly irregular, and the contrast at the border of the kidney is highly variable at the interface of several different tissue types including fluid-filled cysts, calcified cysts, renal parenchyma, and fibrotic tissue. In addition, MR acquisition parameters vary widely from institution, requiring a robust approach which can handle not only the wide range of disease presentations but also the drastic difference in tissue contrast due to how the images were acquired.

We previously developed both semi- and fully automated segmentation approaches to allow accurate and reproducible measurement of TKV in ADPKD patients [24, 25]. Fortunately, these developments have allowed for the creation of a database of thousands of reference standard segmentations by which we have been able to explore novel, next-generation image processing techniques in order to finally and fully address the problem of segmentation of the PKD kidney in order to accurately and reproducibly derive TKV.

We have developed a deep neural network model that can capture both local and global context within the image. This model is based on a convolutional neural network (CNN) approach that performs a series of downsampling (i.e., max pooling operations which select the maximum value from a patch of features which help to reduce the data dimensionality) and upsampling procedures (similar to autoencoders [26], which allow classification to be made at the voxel level). The network also incorporates skip connections (similar to a CNN architecture known as U-Net [27] which connect layers at the same resolution and allow the networks to retain spatial information). The network is a cascade of layers that start by learning low-level features (e.g., edges and lines) and higher-level features (which combine this information to learn what is or is not the kidney). In summary, building a network with these components allows the network to (i) learn both low- and high-order features, (ii) learn both local- and entire image-level context, and (iii) perform voxel-wise classification (i.e., decide whether a voxel belongs to the kidneys or not).

## Method

### MRI Data

Institutional review board approval was obtained for this study. All subjects were appropriately consented for use of bio-sample data for the purpose of identifying methods for improving ADPKD diagnosis and management. De-identified DICOM image data from the TEMPO study [28] was transferred to our institution and converted to the NIFTI file format by the dcm2nii software. The images have a reconstructed matrix size of 256 × 256 × *Z* (with *Z* large enough to cover the full extent of the kidneys within the imaged volume). Image voxel sizes are most commonly on the order of 1.5 mm in-plane with typically 3–4 mm slice thicknesses.

### Reference Standard TKV

The pycysticimage viewer toolkit was used by a trained medical imaging analyst, and the MIROS application was used to create initial kidney segmentations [24]. Afterwards, the segmentations were quality checked and manually corrected when needed. These segmentations were then used with the automated follow-up segmentation approach [25] to generate segmentations for all patient follow-up examinations. These segmentations were also quality checked and manually corrected when needed. The finalized segmentations were used as the reference standard segmentations by which we judged the accuracy of the fully automated approach.

### Deep Learning Model

We developed a convolutional neural network architecture that is based on a semantic segmentation approach. All algorithms were written in Python, with the Keras library and Theano backend. For developing, training, and testing the neural network models, a high-performance GPU workstation (Exxact Corp., Fremont, CA) with 128 Gb of RAM and 4× NVIDIA GeForce GTX 1080, 8 Gb GPUs was used. The network architecture was first optimized on a small subset of the data (*N* = 200 cases). This optimization consisted of extracting 150 cases for training and validation, and then testing on the remaining 50 cases. Exhaustive grid search was then performed to test a range of networks that were shallower and deeper (in terms of layers), thinner and wider (in terms of number and size of kernels), as well as different activation functions (ReLU, tanh). Each network was run for 50 epochs. Based on the best performing network, 11 separate networks were trained (on different data subsets) in order to create an artificial multi-observer deep neural network for fully automated segmentation of polycystic kidneys in MR images. For training and validation, 2000 cases were randomly selected and the networks were each trained on different subsets of the data (80% training, 20% validation split). After training, the remaining 400 cases from those not used for training and validation were used for testing the automated segmentation approach.

### Segmentation Post-processing

Following the segmentation map generated by the deep learning network, a routine to extract the two largest connected components was performed (i.e., the right and left kidneys). This was followed by an active contour and edge detection method in order to finalize the segmentation [24].

### Evaluation of Automated Approach

Comparison statistics were generated from the reference standard segmentations and those made by the automated approach. These comparison statistics included voxel-by-voxel correlation-based metrics and comparison of total volume differences. For the voxel-by-voxel comparisons, a number of commonly used segmentation metrics were calculated. These include the Dice coefficient (or similarity index) that is defined as:

1 $$ \mathrm{Dice}=\frac{2\cdotp \mathrm{TP}}{2\cdotp \mathrm{TP}+\mathrm{FP}+\mathrm{FN}} $$

where TP is true positives (i.e., both reference standard and automated approach classified voxel as being the kidney), FP is false positives (i.e., automated approach falsely classified voxel as being the kidney), and FN are false negatives (i.e., automated approach falsely classified voxel as not being a part of the kidney), and the Jaccard coefficient (or overlap ratio), which is defined as:

2 $$ \mathrm{Jaccard}=\frac{\mathrm{TP}}{\mathrm{TP}+\mathrm{FP}+\mathrm{FN}} $$

Both of these indices vary within the range 0 to 1, where a value closer to 1 indicates a closer similarity between the two segmentations. Sensitivity, specificity, and precision are also reported based on voxel level statistics and the average maximum distance between the borders of the two segmentations was calculated (*D*_mean_). In addition, percent error of TKV as measured by the different approaches was calculated, and Bland-Altman analysis was performed to compare the automated measurement method to the reference standard.

## Results

### Optimized Network Performance

**Fig. 1:**
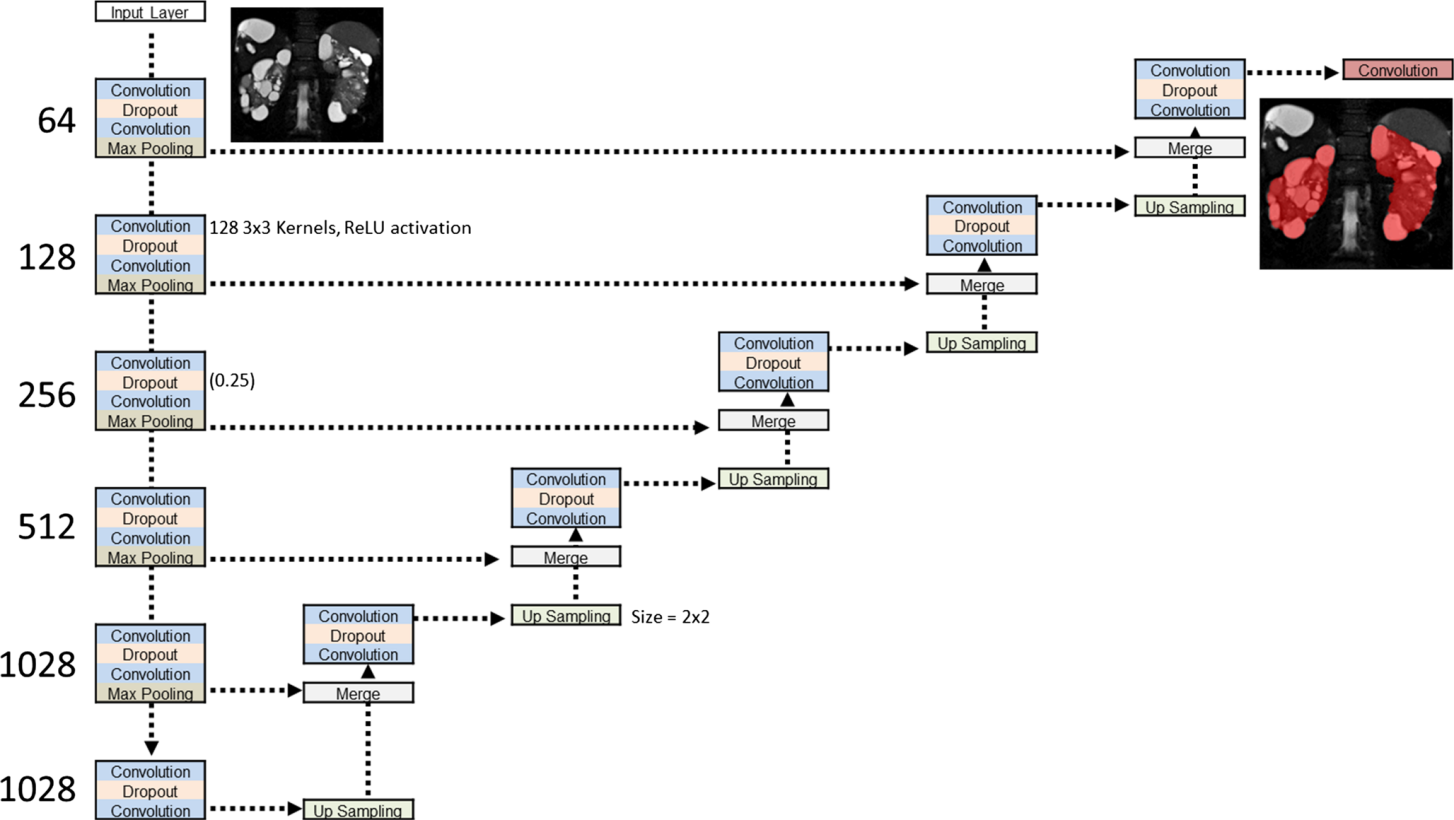
Optimized network architecture consisting of a series of downsampling, upsampling, and skip connections. Each block consists of a series of convolutions (3 × 3 kernels, ReLU activation) and dropout layers (0.35). Both max pooling layers and upsampling layers are of size 2 × 2. The final convolutional layer is a 1 × 1 kernel with sigmoid activation, resulting in classification of each voxel of the input (size 256 × 256)

**Fig. 2:**
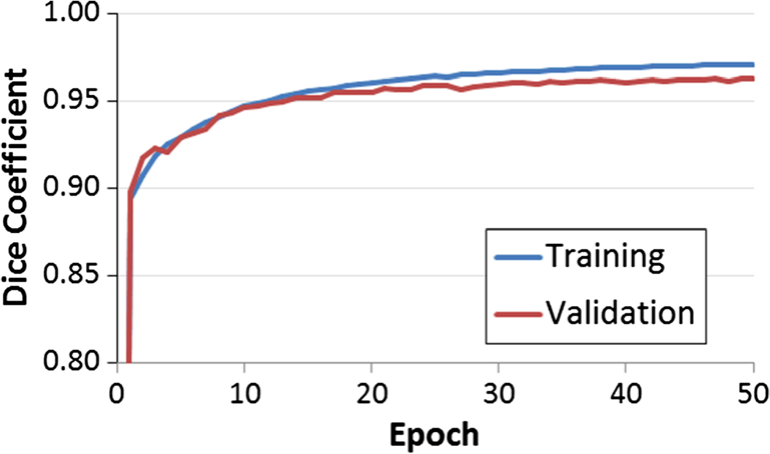
Training and validation curves for the optimized network. Training and validation Dice coefficients of 0.97 and 0.96 were obtained, respectively. Network weights were monitored and saved based on the best performance on validation set

The optimal deep learning network architecture is graphically depicted in Fig. 1 and had a training Dice coefficient of 0.97 and a validation Dice coefficient of 0.96. Shown in Fig. 2 are the training and validation curves for the Dice coefficient calculated at each epoch.

### Artificial Multi-observer Network

Next, 11 of these networks used the 2000 cases for training and validation. Each network was trained on a different subset of the cases. Each network was run for 100 epochs, and the best model was saved based on Dice coefficient. These 11 networks were then used in a majority voting scheme to test their ability to accurately segment the 400 test cases not seen during training and validation.

### Visualization

**Fig. 3:**
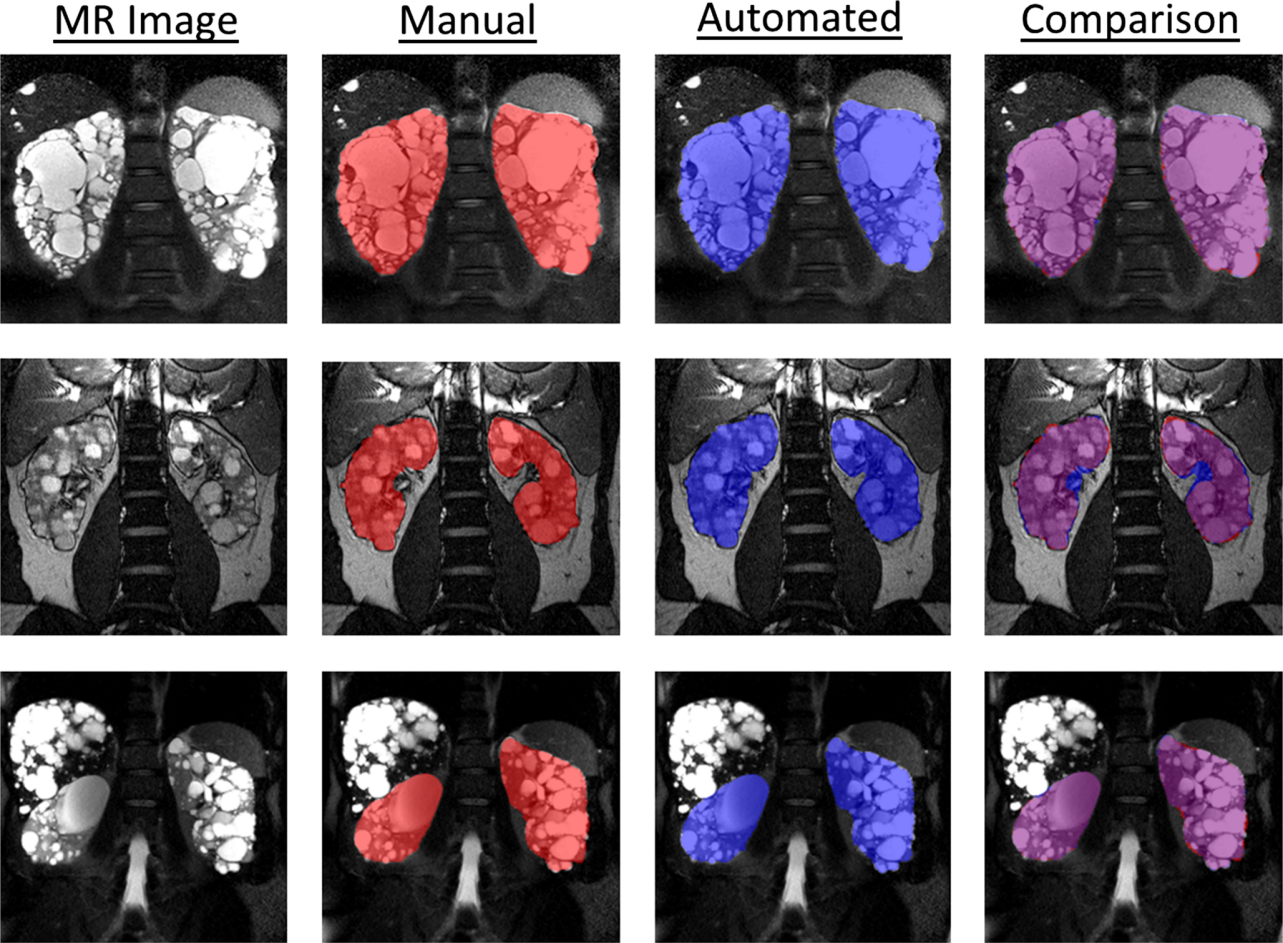
Examples of segmentations obtained for three different patients. Shown in the *left column* are the MR images, the *second column* are the reference standard segmentations, the *third column* are the automated segmentations, and the *right column* are the segmentations overlaid on one another. Reference standard segmentations are shown in *red*, and automated segmentations are shown in *blue*. Regions of overlap are *purple*. Shown in the *top row* is an average example from the dataset, which had a Dice coefficient of 0.96. Shown in the *second row* is the worst-performing case, which had a Dice coefficient of 0.92. The difficulty in this case is the rarer (in terms of this particular dataset) T2-weighted acquisition (a FISP image) which suffers from image artifacts (particularly banding artifacts resulting from intravoxel dephasing). Shown in the *final row* is an example of a patient with significant polycystic liver disease. Notice how the automated approach does not classify the liver, or the liver cysts, as kidney

Visual examples of the result of the multi-observer ensemble method are shown in Fig. 3 along with the reference standard segmentation.

### Similarity Metrics

Table 1 summarizes the similarity statistics for the automated approach compared with the reference standard segmentations. The multi-observer ensemble method had an average percent volume error of 0.68%, a standard deviation of percent volume error of 2.2%, and worst case min = −8.1%, and max = 7.0%. In addition, similarity statistics were as follows: Jaccard = 0.94 ± 0.03, Dice = 0.97 ± 0.01, sensitivity = 0.97 ± 0.02, specificity = 0.99 ± 0.01, and precision = 0.98 ± 0.02 for the unseen test cases.

**Table 1:** Summary statistics for the automated approach compared with the gold standard. Shown are the results for an individual network, as well as the multi-observer approach

Statistic *m* ± SD [min/max]	Individual	Multi-observer
Jaccard	0.93 ± 0.03 [0.78/0.98]	0.94 ± 0.03 [0.85/0.98]
Dice	0.96 ± 0.02 [0.88 0.99]	0.97 ± 0.01 [0.92 0.99]
Sensitivity	0.96 ± 0.02 [0.79/0.99]	0.96 ± 0.02 [0.89/0.99]
Specificity	0.99 ± 0.01 [0.99/1.00]	0.99 ± 0.01 [0.99/1.00]
Precision	0.97 ± 0.02 [0.83/1.00]	0.97 ± 0.02 [0.88/1.00]
*D*_mean_	0.57 ± 0.46 [0.18/4.45]	0.49 ± 0.36 [0.17/3.69]
Volume difference %	−1.42 ± 2.75 [−18.90/15.72]	−0.65 ± 2.21 [−8.06/7.04]

### Automated Measurement of TKV

**Fig. 4:**
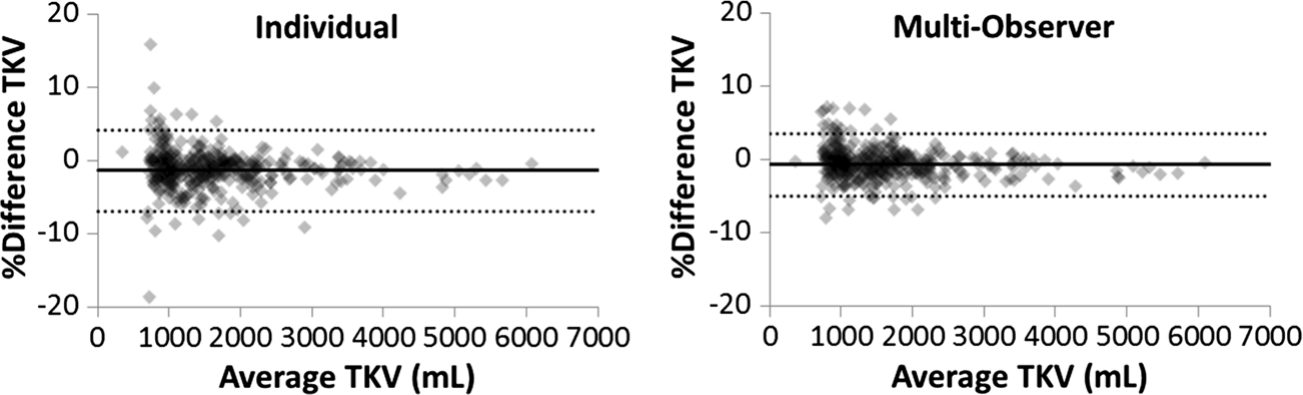
Bland-Altman analysis of the percent difference of TKV measurements obtained by the automated approach and the reference standard segmentations for both an individual network and the simulated multi-observer approach. The mean difference (*solid line*) and 95% confidence intervals (*dotted lines*) are also shown. For the individual network, the *m* ± SD for the percent volume difference was −1.42 ± 2.75 and the 95% confidence intervals were [−6.93 to 4.09]. The *m* ± SD for the percent volume difference was −0.65 ± 2.21 and the 95% confidence intervals were [−4.97 to 3.63]

Shown in Fig. 4 are the Bland-Altman analysis results for an individual network, and the multi-observer ensemble method. For the individual network and the multi-observer ensemble method, the *m* ± SD for the percent volume difference was −1.42 ± 2.75 and −0.65 ± 2.21, respectively.

## Discussion

### Implications for Research and Clinical Trials

High accuracy was obtained by the automated segmentation approach and performance on a level comparable to two different people performing segmentations (interobserver variability) was achieved (comparing the automated approach to the results generated manually). The combination of high accuracy without the necessity of human interaction is an important advance for both the clinical practice and research trials. In the case of research trials, the ability to efficiently and objectively detect small changes reduces the cost of performing a study and results in a much more rapid decision about a drug’s effectiveness. This current study can work harmoniously with our previous work for establishing a baseline measurement [24, 25], and automatically performing a reread of subsequent scans in the same patient [24, 25].

Our automatic segmentation approach offers a fast and accurate method to measure the TKV imaging biomarker for patients with diseased kidneys. This automation allows for robust study repeatability and removal of user bias in segmentations and measurement of TKV. The automatic segmentation has useful clinical applications such as following progression of the disease as well as judging the effectiveness of interventions. Once the network is trained, the automated approach segmentations are computed in the matter of minutes, whereas manual segmentations take 45–90 min. Thus, our method could enable the routine clinical use of TKV data.

An important strength of the developed approach is the success that was observed in terms of accurately handling liver cysts and major vasculature (e.g., renal artery and vein). This differentiation is a difficult task for humans and it appears that there are clearly identifiable imaging features that were derived that allowed the automated approach to successfully differentiate not only the liver from the kidney but also adjacent liver cysts from those pertaining to the kidney.

Lastly, having the ability to accurately and reproducibly segment the PKD kidney not only allows for measurement of TKV but also allows characterization of additional imaging biomarkers, such as calculating cystic burden or describing the “class” of cystic distribution [29], calculating imaging texture features [30], or measuring parameters derived from quantitative MRI acquisitions [31].

### Limitations

While the developed approach appears very promising, there exist some limitations that may still require a final quality check by a trained imaging analyst. For instance, renal pelvis delineation appears highly variable. This we attribute to the known high variability of human readers in performing this task. Fortunately, the fact that an automated approach will come to the same conclusion every time will be a helpful step towards improving the reproducibility of TKV measurements. In addition, being able to simulate the results obtained from multiple people performing the segmentations removed outlier cases and resulted in a much more consistent and reproducible measurement of TKV.

## Conclusion

We obtained high-quality segmentations of severely diseased organs matching human performance with a fully automated computer algorithm which simulates a multi-observer majority voting scheme. This method should be further explored for its utility in research studies and the clinical practice.
